# Nutritional risk and its relationship with physical function in community-dwelling older adults

**DOI:** 10.1007/s40520-022-02171-3

**Published:** 2022-07-01

**Authors:** Ilse Bloom, Jean Zhang, Camille Parsons, Gregorio Bevilacqua, Elaine M. Dennison, Cyrus Cooper, Kate A. Ward

**Affiliations:** 1 MRC Lifecourse Epidemiology Centre, University of Southampton, Southampton, SO16 6YD UK; 2 NIHR Southampton Biomedical Research Centre, University of Southampton and University Hospital Southampton NHS Foundation Trust, Southampton, SO16 6YD UK; 3 Victoria University of Wellington, Wellington, New Zealand; 4 NIHR Musculoskeletal Biomedical Research Unit, University of Oxford, Oxford, OX3 7LD UK

**Keywords:** Community, Nutritional risk, Older adults, Physical function, Screening tool

## Abstract

**Background:**

Malnutrition is a serious concern in older populations. Simple screening approaches are needed to identify signs of early nutritional risk in older people, to allow intervention before overt malnutrition develops, along with the poorer health outcomes associated with it, such as sarcopaenia and frailty. The main aim of this study was to compare nutrition risk scores, calculated from the DETERMINE Checklist (‘Determine Your Nutritional Health’, also known as the Nutrition Screening Initiative Checklist), with physical function variables in a group of community-dwelling older adults. Another aim was to assess the prevalence of nutrition risk using the DETERMINE and the MUST (Malnutrition Universal Screening Tool).

**Methods:**

Participants of the Hertfordshire Cohort Study (HCS) were recruited and visited at home by a trained researcher. Self-reported physical function was assessed using the SF-36 PF (Short Form-36 Physical Function) scale. The Short Physical Performance Battery (SPPB) was performed, which included the assessment of gait speed, chair rise time and standing balance. Handgrip strength was measured using a Jamar dynamometer. Frailty was assessed according to the presence of at least three of the following Fried frailty criteria: unintentional weight loss, weakness, self-reported exhaustion, slow gait speed and low physical activity. Nutrition risk scores were calculated from the DETERMINE checklist (range 0–21). Nutritional risk was also assessed using the MUST. Analyses were adjusted for sex, age, age left education and number of comorbidities.

**Results:**

In the study, 176 participants (94 men and 82 women), median age 83.3 (IQR 81.5–85.7) years, were assessed. Almost half (47%) scored either ‘moderate’ (score 3–5) or ‘high’ (score ≥ 6) nutritional risk (9% were at high risk), using the DETERMINE checklist, whereas 8% were at risk using the MUST. Higher nutrition risk scores, calculated from DETERMINE, were associated with poorer self-reported physical function (difference in SF-36 PF score: − 0.36, 95% CI (− 0.60, − 0.12) SD per unit increase in nutrition risk score, *P* = 0.004) and higher odds of being frail (odds ratio Fried frailty: 2.23, 95% CI (1.15, 4.33), *P* = 0.017). There were no significant associations between DETERMINE nutrition risk scores and the other variables examined.

**Conclusion:**

Cross-sectional associations between higher nutrition risk scores, assessed from the DETERMINE checklist, and poorer self-reported physical function and greater likelihood of frailty suggest that this screening tool may have utility for screening older populations. Prospective studies are required to explore the ability of the tool to predict poor physical function and frailty, though these data suggest it has potential for early, simple detection of nutritional problems in community-living older adults.

## Introduction

Malnutrition is a common problem in older populations across Europe, including in the UK, where over a million people over 65 years have been estimated to be affected by it, the majority of whom are living in the community [1–3]. Malnutrition in older people is a serious concern both in terms of individual health, well-being and quality of life, and in terms of the burden it places on health and social care systems [4, 5]; in England alone, the total health and social care expenditure associated with malnutrition was estimated to be £19.6 billion in 2011–2012, with about half of this expenditure for older people (aged over 65 years) [4]. Malnutrition in older people is associated with poorer functional health, including sarcopaenia and frailty [5–7].

Nutritional screening at an early stage and appropriate intervention could be key to the prevention of malnutrition [5, 8]. The need for further implementation of screening, particularly in the community setting, has been previously highlighted [8, 9]. Various barriers to screening for malnutrition have been reported, including limited access of healthcare professionals to individuals at risk of malnutrition in the community [9]. Nutritional screening tools, which do not require specialist knowledge to use and identify risk factors that may lead to the development of malnutrition, could help to identify a need for further assessment, and thus enable intervention before the development of overt malnutrition, or any further deterioration in nutritional status.

The ‘Determine Your Nutritional Health’ (DETERMINE) screening tool was developed in the USA to identify nutritional problems in older populations, and was designed for self-completion, requiring no specialist knowledge or equipment [10]. The potential utility of the DETERMINE checklist as a screening tool for use in the community setting has been highlighted; however, further studies are needed to assess its validity for identifying nutrition risk in older adults [8, 11]. Although its predictive validity has been mixed in relation to outcomes such as mortality [12–14], accumulating evidence suggests the potential of this tool for the prediction of outcomes related to physical function and independence [15–17]. The MUST (Malnutrition Universal Screening Tool) is the most commonly used screening tool by healthcare professionals in the UK to screen for malnutrition in adults [18]. This tool has been designed for application to all adults, across all healthcare settings, as well as in the community, and focuses on identifying risk of malnutrition per se, particularly the detection of poor protein–energy status [8, 18]. This contrasts with the DETERMINE tool, which was designed for use specifically in older adults living in the community, and focuses on identifying general risk factors for poor nutrition in older age [8]. This study aimed to compare nutrition risk scores, calculated from the DETERMINE checklist, with physical function variables in a group of community-dwelling older adults. Another aim of this study was to assess the prevalence of nutrition risk using two nutritional screening tools: the DETERMINE checklist and the MUST.

## Methods

Participants were recruited from the Hertfordshire Cohort Study (HCS), an established birth cohort study of men and women born between 1931 and 1939 in the county of Hertfordshire, UK [19, 20]. Between November 2019 and March 2020, 176 participants were visited at home by a trained researcher who administered a questionnaire that included information on medical history, medication use, lifestyle and social and psychological factors. The study received ethical approval from the East of England—Cambridgeshire and Hertfordshire Research Ethics Committee, reference number 11/EE/0196. All participants gave written informed consent before participating in this study.

At the visits, height was measured to the nearest 0.1 cm using a portable, free-standing stadiometer (Harpenden Pocket Stadiometer, London, England). Body weight was measured to the nearest 0.1 kg, with the participant wearing clothes but not shoes, using portable SECA digital scales (Model 835). BMI (kg/m^2^) was calculated (weight (kg)/ height (m)^2^).

Self-reported physical function was assessed using the SF-36 PF (Short Form-36 Physical Function) scale [21]. At the visits, various measurements were also performed, including the Short Physical Performance Battery (SPPB) test, which included the assessment of gait speed, standing balance and sit-to-stand performance (chair stand test) [22, 23]. Gait speed was measured using an eight-foot course, with participants being asked to walk at their usual pace while being timed using a stopwatch; participants could make use of assistive devices, such as canes, if necessary. Gait speed was calculated by dividing the distance walked by the time between the first and last step. For the chair stand test, participants were asked to move from a sitting position to a fully upright standing position five times as quickly as possible, with their arms crossed across their chest, while being timed from their initial sitting position until upright on the fifth repetition. The standing balance test involved a semi-tandem stand where participants were asked to place one foot in front of the other such that the big toe of one foot was touching the side of the heel of the other. If participants could not hold the semi-tandem stand for 10 s, they were asked to perform a side-by-side stand (standing with feet side by side). If they could hold the semi-tandem stand for 10 s, they were also asked to attempt a full tandem stand where they placed one foot in front of the other (touching heel to toe) and held this position for as long as they were able, up to 10 s. A physical performance score was derived from the above three tests, according to the SPPB scoring guidelines [23]. Participants who could not complete either the gait speed test or the chair rise test were given a score of 0. The remaining participants’ times were divided into quartiles and scored 1–4, the slowest to the fastest quartile. For the standing balance test, if participants could maintain balance in the tandem stand for at least 10 s, a score of 4 was given; if their time was ≥ 3 and < 10 s, they scored 3; if they maintained balance for < 3 s but were able to maintain a semi-tandem stand, they scored 2; if they were unable to perform the semi-tandem stand but could perform the side-by-side stand, they scored 1; and if they could do neither the semi-tandem nor the side-by-side stand, they scored 0. The scores for the three tests were then summed, with a maximum possible score of 12 and a minimum of 0. Scores of 9 or lower were considered to be indicative of poor physical performance.

Handgrip strength was measured using a handgrip Jamar dynamometer, three times for each hand and the maximum value was used for analysis [24]. Frailty was defined as the presence of three or more of the following Fried frailty criteria [25]: unintentional weight loss, weakness, self-reported exhaustion, slow gait speed and low physical activity. Weight loss was assessed by asking whether the participant had lost any weight unintentionally in the preceding 3–6 months; answering affirmatively was considered as unintentional weight loss. Weakness was defined as a handgrip strength of < 27 kg for men and < 16 kg for women [7]. Exhaustion was assessed by asking the participant how often in the preceding week they felt that “everything they did was an effort” or that “they could not get going”. Participants who reported feeling this way for either ‘a moderate amount of time’ or ‘most of the time’ were categorised as ‘exhausted’. Slow gait speed was defined as a gait speed of ≤ 0.8 m/s. Physical activity was assessed by the average amount of time (in minutes per day) spent walking outside, cycling, gardening, playing sports or doing housework in the preceding 2 weeks [26]. Low physical activity was defined as an activity time in the bottom fifth of the sex-specific distribution (≤ 58 min/day for men and ≤ 90 min/day for women).

Nutrition risk scores were calculated using the DETERMINE checklist [10]. This tool includes ten questions on age-related and contextual factors that are linked to poor nutrition in older age: illness leading to dietary changes; eating few meals/reduced appetite; eating few fruits, vegetables or milk products; high alcohol intake; eating difficulties due to tooth or mouth problems; not having enough money for food; eating alone; frequent medication usage; unintentional weight change; and physical difficulties with shopping, cooking or eating. Responses are weighted to calculate an overall nutrition risk score for each participant, by summing the ten scored items, with thresholds given to identify categories of risk: ‘low’ (0–2), ‘moderate’ (3–5) and ‘high’ (≥ 6) nutritional risk; total nutrition risk scores range from 0 to 21 [10]. Nutritional risk was also assessed using the MUST, which includes three scores: body mass index (BMI) (BMI ≤ 20 kg/m^2^ indicates risk), unintentional weight loss (unintentional weight loss during the preceding 3–6 months; ≥ 5% indicates at risk) and an acute disease effect score. From this information, total MUST scores are calculated and grouped into three risk categories: low risk (score 0), medium risk (score 1) or high risk (score ≥ 2) [18, 27].

### Statistical analysis

Descriptive characteristics are given as mean with standard deviation (SD) for continuous normally distributed variables, median with interquartile range (IQR) for continuous variables with a skewed distribution, or counts and percentages for categorical variables, as appropriate.

The calculated nutrition risk score from the DETERMINE checklist was used as a continuous variable in regression analyses. The relationships between the nutrition risk score and gait speed, chair rises time, physical performance (SPPB) score, SF-36 physical functioning score, and grip strength were examined using multivariate linear regressions. Fisher–Yates rank-based inverse normal transformations were performed to create z-scores (FY *z* scores) to enable the comparison of effect sizes. The associations between the nutrition risk score and Fried frailty, tandem stand < 10 s, and low SPPB score (≤ 9) were examined using multivariate logistic regressions. Analyses were performed with adjustments for sex, age, age left education and number of comorbidities. Number of comorbidities was assessed by asking whether the participant had been told by a doctor that they had any of the following conditions: high blood pressure, diabetes, lung disease, rheumatoid arthritis, multiple sclerosis, cancer, vitiligo, depression, Parkinson’s disease, heart disease, peripheral arterial disease, osteoporosis, thyroid disease and stroke. Analyses were performed using Stata version 16.

## Results

The study included 176 participants, 94 (53%) men and 82 (47%) women, with a median age of 83.3 years (IQR 81.5–85.7 years). Table 1 shows the descriptive characteristics of the whole group, and according to category of nutritional risk, assessed from DETERMINE. Regarding their marital status, 107 (61%) participants were married, with the remainder either widowed (*n* = 53; 30%), single (*n* = 9; 5%) or divorced/separated (*n* = 7; 4%). Most participants (*n* = 108; 62%) had never smoked, while 63 (36%) were former smokers and 4 (2%) current smokers. The median nutrition risk score was 2 (IQR 1–4). As there were no significant gender interactions with nutrition risk scores, pooled analyses were carried out (for the interaction analysis a statistical significance value of 5% was used).

**Table 1:** Characteristics of the study participants (*n* = 176; *n* = 94 (53%) men and *n* = 82 (47%) women), for the whole group and by category of nutritional risk

	Nutritional risk												
	All			Low (score 0–2)			Moderate (score 3–5)			High (score ≥ 6)			*P* value^1^
	*N*	Mean	SD	*N*	Mean	SD	*N*	Mean	SD	*N*	Mean	SD	
Height (cm)	175	165	9	92	164	9	67	166	9	16	166	11	0.31
Weight (kg)	173	73.8	13.4	93	73.6	12	65	74.8	14.6	15	70.5	16.6	0.53
BMI (kg/m^2^)	172	27.1	4.0	92	27.4	3.7	65	27.1	4.4	15	25.1	3.7	0.13
Grip strength (kg)	165	27.0	9.2	89	27.3	9.6	62	26.1	8	14	28.7	11.7	0.58
Gait speed (m/s)	166	0.62	0.18	90	0.62	0.18	62	0.64	0.15	14	0.6	0.31	0.74
Prudent diet score^2^	162	− 0.06	1.45	87	0.02	1.55	61	− 0.08	1.29	14	− 0.48	1.49	0.48

	*N*	Median	IQR	*N*	Median	IQR	*N*	Median	IQR	*N*	Median	IQR	
Age (years)	176	83.3	81.5–85.7	93	83	81.4–85.4	67	83.6	82.0–85.9	16	84.1	81.8–85.6	0.74
Activity time in last 2 weeks (min/day)^3^	171	126	75–176	91	141.4	84.3–231.4	64	116.3	61.1–157.1	16	111.1	86.8–153.2	**0.05**
Number of comorbidities	176	2	1–3	93	2	1.0–2.0	67	2	1.0–3.0	16	2	1.5–2.5	**0.04**
Number of medications	176	5	3–7	93	4	2.0–7.0	67	6	4.0–8.0	16	5.5	4.0–8.0	**0.02**
Chair rises time (s)	151	13.2	11.1–15.5	84	12.9	11.0–15.5	57	13.4	11.6–16.0	10	12.8	10.8–14.6	0.67
Physical performance score (SPPB)	167	8	6–10	91	8	6.0–11.0	62	8	7.0–10.0	14	6.5	5.0–11.0	0.77
SF-36 Physical functioning score	176	75	50–90	93	80	60.0–95.0	67	75	50.0–90.0	16	52.5	27.5–72.5	**0.01**

	Total *N*	*N*	%	Total *N*	*N*	%	Total N	*N*	%	Total *N*	*N*	%	
Living alone	176	60	34.1	93	23	24.7	67	28	41.8	16	9	56.3	**0.04**
Unintentional weight loss^4^	171	26	15.2	92	8	8.7	63	13	20.6	16	5	31.3	**0.02**
Tandem stand < 10 s	139	44	31.7	77	25	32.5	50	14	28.0	12	5	41.7	0.62
Low SPPB score (≤ 9)	167	114	68.3	91	62	68.1	62	44	71.0	14	8	57.1	0.60
Fried frailty	162	25	15.4	88	9	10.2	60	11	18.3	14	5	35.7	**0.03**

The DETERMINE checklist was used to derive a nutrition risk score; participant characteristics are shown according to the published thresholds to categorise different levels of risk [10]

^1^Unadjusted *p* value for trend across the continuous nutrition risk score variable

^2^Diet was assessed using a short food frequency questionnaire; derived ‘prudent’ diet scores described diet quality (higher scores indicate greater consumption of fruits, vegetables, wholegrain cereals and oily fish, and lower consumption of white bread, added sugar, full-fat dairy products, chips and processed meat) [28]

^3^Physical activity was assessed by the average amount of time (in min/day) spent walking outside, cycling, gardening, playing sports or doing housework in the preceding 2 weeks [26]

^4^Any self-reported unintentional weight loss in the preceding 3–6 months

Almost half (47%) of participants scored either ‘moderate’ (score 3–5) or ‘high’ (score ≥ 6) nutritional risk, using the DETERMINE checklist; 67 (38%) were at moderate risk and 16 (9%) were at high risk, with 93 (53%) at low nutritional risk. The overall prevalence of nutritional risk (and in this case, malnutrition risk) assessed using the MUST was 8%, including 5% at medium risk and 3% at high risk (Table 2). Table 2 shows the overlap between categories of risk, for both the DETERMINE and the MUST.

**Table 2:** Cross-tabulation of prevalence of nutritional risk according to two different screening tools: DETERMINE and MUST (cell = *N*; % (of total))

MUST				
DETERMINE	Low risk	Medium risk	High risk	Total
Low nutritional risk	89	2	0	91
	53.9%	1.2%	0%	55.2%
Moderate nutritional risk	52	4	3	59
	31.5%	2.4%	1.8%	35.8%
High nutritional risk	11	2	2	15
	6.7%	1.2%	1.2%	9.1%
Total	152	8	5	165
	92.1%	4.9%	3%	100%

Participants with higher nutritional risk tended to be less active, to have a greater number of comorbidities and take a greater number of medications (Table 1). There was a strong cross-sectional association between higher nutritional risk and living alone; 56% (*n* = 9) of those at high nutritional risk were living alone, compared with 25% (*n* = 23) of those at low risk (Table 2). Univariate cross-sectional analyses also showed a higher nutrition risk score to be associated with poorer self-reported physical function, greater unintentional weight loss (31% of participants at high nutritional risk reported unintentional weight loss, compared with 9% of those at low risk) and frailty (36% of those in the high nutritional risk group were ‘frail’, compared with only 10% of those at low risk) (Table 1).

Table 3 shows the numbers and proportions of participants who responded affirmatively to each of the individual DETERMINE checklist components. Almost three-quarters (73%) of participants reported taking three or more different prescribed or over-the-counter medications per day; over a third (35%) reported eating alone most of the time; over a quarter (27%) reported having an illness or condition that made them change the kind and/or amount of food that they ate; and almost a fifth (18%) of participants reported eating few fruits or vegetables or milk products. Other commonly reported components were unintentional weight loss or gain (12.5%); having tooth or mouth problems making it difficult to eat (8%); and not always being physically able to shop, cook and/or feed themselves (7%). The least commonly reported items were eating fewer than two meals per day (3%) and having three or more alcoholic drinks almost every day (2%), while not having enough money to buy food was not reported in this group.

**Table 3:** Number (%) of participants who answered ‘yes’ to each of the DETERMINE components

Item		*N* (%)
1	I have an illness or condition that made me change the kind and/or amount of food I eat	48 (27.3%)
2	I eat fewer than two meals per day	5 (2.8%)
3	I eat few fruits or vegetables or milk products	32 (18.2%)
4	I have three or more drinks of beer, liquor or wine almost every day	4 (2.3%)
5	I have tooth or mouth problems that make it hard for me to eat	14 (8.0%)
6	I do not always have enough money to buy the food I need	0 (0%)
7	I eat alone most of the time	62 (35.2%)
8	I take three or more different prescribed or over-the-counter drugs a day	129 (73.3%)
9	Without wanting to, I have lost or gained 10 pounds in the last 6 months	22 (12.5%)
10	I am not always physically able to shop, cook and/or feed myself	12 (6.8%)

Table 4 shows the associations between the DETERMINE nutrition risk score and physical function variables. After adjusting for sex, age, age of leaving education and number of comorbidities, higher nutrition risk scores were associated with poorer self-reported physical function (SF-36 PF score: − 0.36, 95% CI (− 0.60, − 0.12) SD per unit increase in nutrition risk score, *P* = 0.004) and higher odds of being frail (odds ratio Fried frailty: 2.23, 95% CI (1.15, 4.33), *P* = 0.017). There were no significant associations between nutrition risk scores and the other physical function variables examined, i.e. gait speed, chair rises time, standing balance, physical performance score (SPPB score), or grip strength.

**Table 4:** Associations between the continuous DETERMINE nutrition risk score and physical function variables

Variables	*N*	Fully adjusted*	
		Regression coefficient (95% CI)	*p* value
Gait speed (FY z-score)	166	0.06 (− 0.17, 0.28)	0.619
Chair rises time (FY z-score)	151	− 0.04 (− 0.29, 0.21)	0.745
Physical performance (PP) score (FY z-score)	167	− 0.05 (− 0.29, 0.20)	0.710
Grip strength (FY z-score)	165	− 0.09 (− 0.25, 0.07)	0.276
SF-36 Physical functioning score (FY z-score)	176	− **0.36 (**− **0.60, **− **0.12)**	**0.004**

	*N*	Odds Ratio (95% CI)	*p* value
Fried frailty	162	**2.23 (1.15, 4.33)**	**0.017**
Tandem stand < 10 s	139	1.28 (0.71, 2.30)	0.416
Low SPPB score (≤ 9)	167	0.79 (0.45, 1.36)	0.388

*CI* confidence interval, *FY* Fisher–Yates

*Adjusted for sex, age, age left education and no. of comorbidities (self-reported number of doctor-diagnosed comorbidities)

Significant associations (*p* < 0.05) are highlighted in bold

Further analyses that examined associations between the nutrition risk score and other variables also showed no significant associations, in the fully adjusted model: diet quality (prudent diet score) (− 0.11, 95% CI (− 0.35, 0.12) SD); BMI (− 0.20, 95% CI (− 0.43, 0.03) SD); and unintentional weight loss (OR 0.92, 95% CI (0.28, 1.56)).

## Discussion

This study used the DETERMINE nutrition screening tool to identify nutritional risk and assessed its relationship with physical function variables in a group of community-dwelling older adults in the UK. This cross-sectional study has shown that greater nutritional risk, calculated from the DETERMINE checklist, was associated with poorer self-reported physical function and higher likelihood of frailty, but not with other physical function variables, namely gait speed, chair rises time, standing balance, SPPB score or grip strength.

The absence of association between the DETERMINE nutrition risk score and grip strength or diet quality contrasts with some of the findings from our previous study [15]. We did, however, find the nutrition risk score to be associated with poorer self-reported physical function and higher odds of being frail. Frailty is a multifaceted geriatric syndrome that is characterised by a decline in multiple physiological systems or functions, and has been shown to predict adverse health outcomes, including disability, reduced quality of life and mortality [7, 25]. Our findings are consistent with those from other studies in older people from the USA, Japan and Singapore that have indicated the predictive utility of this tool for outcomes related to independence and functional capacity; higher nutritional risk assessed with DETERMINE was negatively associated with living independently in the community [16]; high nutrition risk was found to be associated with functional decline in both activities of daily living (ADL) and instrumental ADL (IADL) over 2 years [17], and both moderate and high nutritional risk were related to frailty [29]. A recent study of community-living older adult in Singapore found that change in nutritional risk assessed using DETERMINE, specifically decrease in risk from moderate or high nutritional risk to low risk, was associated with lower incidence of IADL or ADL disability [30]. The study also found that an increase in nutritional risk using DETERMINE was associated with increased risk of mortality, and that persistent nutritional risk (moderate or high nutritional risk) was associated with higher incidence of poor quality of life and mortality [30].

We found a similar proportion of older adults categorised as being at either moderate or high nutritional risk (47%) as reported in our previous study in slightly younger (mean age 78 (SD 8) years) community-dwelling UK older adults (53%) [15]. However, in the present study the proportion at high risk was considerably lower (9%) than in the previous study (17%) [15]. It is possible that despite the present study population being older on average, participants might have been in relatively better overall health (for example, they had on average two comorbidities, compared to more than four comorbidities in the previous study).

The most commonly reported components from the DETERMINE checklist in this study were frequent/high medication usage (73% of participants reported taking ≥ 3 different medications per day), eating alone (35% ate alone most of the time) and having an illness that has led to dietary changes (27% had an illness or condition that influenced dietary intake), which suggests that these may be important nutritional risk factors in community-living older people. These findings are in line with those of Katsas and colleagues from an older population in Greece, although tooth or mouth problems were more prevalent in this study [31]. It is likely that chronic illness, comorbidities and increased use of medications may contribute to a reduction in appetite and affect dietary intake in older age [32]. Our findings suggest that these factors (eating alone, medication usage and illnesses leading to dietary changes) might be the main risk factors driving the associations between greater nutritional risk and poorer self-reported physical function and greater likelihood of frailty reported in this study. However, it should be noted that these associations remained robust to adjustment for self-reported total number of comorbidities.

The present study also examined the prevalence of nutrition risk assessed using the DETERMINE checklist and the MUST. While a higher nutrition risk score calculated using the DETERMINE checklist indicates greater presence of factors that are linked to poor nutrition in older age, a higher score calculated from the MUST indicates low body mass and/or unintentional weight loss. The prevalence of malnutrition risk using the MUST was 8%, which is comparable to that found in a recent study of community-living older adults in the UK (10%) [33]. Notably, in the present study, DETERMINE identified an additional 39% people at risk (47% of participants were either at moderate or high nutritional risk), compared to the MUST. This could indicate that the DETERMINE tool might be more discriminatory than MUST in detecting earlier signs of nutritional risk, before there is substantial unintentional weight loss or reductions in body weight. Our findings are consistent with those of Borkent and colleagues, in their study comparing two different screening tools in the community setting, in which they highlighted the distinction between screening tools for nutrition risk and for overt malnutrition [34]. It is likely that the two tools examined in our study measure different aspects of nutritional risk. The MUST tool, in similarity with most screening tools that have been validated in older adults, focuses on identifying risk of protein-energy malnutrition [34]. Conversely, DETERMINE is one of a few tools that focuses on identifying other aspects of nutrition risk, including risk factors for nutritional problems in later life, and could thus be used to complement the MUST, in community-living older people [8, 34]. DETERMINE is a simple nutrition checklist that could be used as an initial screening step, by health and social care professionals, or indeed by older adults or their carers themselves.

Limitations of this study include its cross-sectional design, with physical function variables only assessed at one time point and physical tests only performed on one day, which may not capture the usual performance. In this study, we did not consider the potential impact of mood on the self-report of physical function-related parameters and symptoms (such as the report of SF-36 PF scale items and the self-reported components of Fried frailty). Although this study found associations between greater nutritional risk (calculated from the DETERMINE checklist) and poorer self-reported physical function and higher likelihood of frailty, unexpectedly, there were no associations with objectively measured physical function variables, i.e. gait speed, chair rises time, standing balance, SPPB score and grip strength. The SF-36 PF scale includes questions on the extent to which a participant’s health limits them in various daily activities, including vigorous activities, moderate activities (e.g. pushing a vacuum cleaner), lifting or carrying groceries, climbing stairs, bending, kneeling or stooping, walking and bathing or dressing themselves. The Fried frailty criteria include three self-reported components, namely asking participants about unintentional weight loss, feelings of exhaustion and about time spent engaging in various activities, to assess low physical activity. Thus, our findings appear to suggest greater ability of the DETERMINE tool to detect these subjective physical symptoms/difficulties, but less sensitivity to identify other aspects of poorer physical performance measured objectively. Another limitation is that the subset of HCS participants studied may not be representative of the wider population of community-living older people, and therefore the prevalence of high nutritional risk reported may not be generalisable. The strengths of this study include the objective measures of physical function, with tests performed in participants’ own homes and administered by trained researchers.

## Conclusion

Efforts to prevent malnutrition need to be taken as early on as possible to ensure better health outcomes for older people. Screening tools that are simple, implementable and practical for use without specialist knowledge are not widely available in community settings. Given that most older people affected by malnutrition are living in the community, the use of a tool such as the DETERMINE that detects factors that are associated with malnutrition risk in a simple way could be a valuable first step in the prevention of malnutrition.

While our previous study [15] used a checklist adapted from the DETERMINE to assess nutritional risk in UK older adults recruited from outpatient clinics, the complete tool had not yet been tested in community-dwelling otherwise healthy older people in the UK. This study examined nutritional risk, assessed using DETERMINE, in relation to a comprehensive battery of musculoskeletal functional ability measures. We report cross-sectional associations between higher nutrition risk scores, assessed from the DETERMINE checklist, and poorer self-reported physical function and greater likelihood of frailty. Furthermore, this is the first time that prevalence of nutrition risk assessed using the DETERMINE checklist has been compared to the more complex MUST tool.

This simple screening tool detected a range of nutritional risk factors in community-living older people and produced risk scores that were associated with clinically important outcomes. It could be used as an important initial tool to inform further assessment by health and social care professionals for provision of appropriate support to address nutritional risk in a timely manner.
